# Closed atrium bipolar radiofrequency box lesion for concomitant surgical atrial fibrillation ablation

**DOI:** 10.3389/fcvm.2025.1655695

**Published:** 2025-08-20

**Authors:** Simon Pecha, Johannes Petersen, Yalin Yildirim, Ilia Bazhanov, Hermann Reichenspurner, Yousuf Alassar

**Affiliations:** ^1^ Department of Cardiovascular Surgery, University Heart Center Hamburg, Hamburg, Germany; ^2^ DZHK, Hamburg, Germany

**Keywords:** atrial fibrillation, surgical ablation, arrhythmia surgery, left-atrial box lesion, AF surgery

## Abstract

**Background:**

In patients with atrial fibrillation (AF) undergoing coronary artery bypass grafting (CABG) or aortic valve replacement (AVR), many surgeons are reluctant to open the left atrium for surgical ablation. However, especially in those with persistent AF, a box lesion isolating the entire posterior left atrial wall may be beneficial. Here, we describe our initial experience with a novel closed atrium bipolar radio-frequency left atrial box ablation technique.

**Methods:**

Between January 2023 and June 2024, 22 patients underwent the closed atrium radio-frequency box lesion set. Left left atrial appendage (LAA) closure was performed using an LAA clip in all patients. We evaluated the technical feasibility, safety, and efficacy of this new concomitant surgical AF ablation approach.

**Results:**

The mean patient age was 67.9 ± 5.3 years, and 68.2% were male. 12 patients (54.5%) had persistent AF, while 10 (45.5%) had paroxysmal AF. Creation of a complete box lesion from the right side was feasible in 14 patients; in 8 patients, the lesion had to be completed from the left side. No major ablation-related complications occurred. Successful intraoperative LAA closure was confirmed by TEE in all patients. There were no periprocedural strokes, and in-hospital mortality was 0%. Freedom from AF was 86.4% at discharge and 77.2% at a mean follow-up of 12.6 ± 3.9 months.

**Conclusion:**

The closed atrium left atrial box lesion technique for surgical treatment of AF concomitant with CABG or AVR is safe and technically feasible. This approach enables complete isolation of the posterior left atrial wall without the need to open the left atrium.

## Introduction

Atrial fibrillation (AF) stands as the most prevalent arrhythmia and is linked to thromboembolic incidents, such as strokes, and is even correlated with an increase in mortality (1, 2). Additionally, it contributes to an increased frequency of hospitalizations and can lead to heart failure (1, 2). Concomitant Surgical AF ablation has demonstrated restoration of sinus rhythm in both, retrospective and prospective randomized trials (3–7). Retrospective studies and prospective registries have also indicated a survival advantage for patients with atrial fibrillation undergoing concurrent AF surgery (5, 8, 9). Consequently, guidelines and consensus statements recommend concurrent surgical ablation for atrial fibrillation (10, 11). In recent ESC/EACTS guidelines, surgical ablation in non-mitral valve surgery has a class IIa indication.

The original Cox Maze procedure, incorporating the cut-and-sew technique, has evolved into the Cox Maze III procedure, serving as the gold standard for surgical ablation for many years. However, due to the complexity of this technique, it has been employed by only a limited number of surgeons. The replacement of the cut-and-sew technique with the creation of thermal lesions has simplified the procedure, leading to widespread application as the Cox MAZE IV procedure. Throughout the years, numerous modifications of the original Cox Maze IV lesion set have been utilized with varying success rates. In procedures where the atria are not routinely opened for the surgical process, such as in CABG or AVR cases, many surgeons are hesitant to perform an atriotomy to conduct a complete left-atrial or even a biatrial ablation. However, electrical isolation of the posterior left atrial wall has been shown to increase success rate of AF ablation in prior studies. We here describe and evaluate a new technique of closed atrium left-atrial box lesion for surgical ablation in patients undergoing AVR or CABG procedures.

## Materials and methods

Between January 2023 and December 2024, 22 patients underwent concomitant surgical ablation with the new closed atrium left-atrial box lesion set in our institution. IRB approval was obtained (Ethikkommission der Ärztekammer Hamburg 2020-10183). A retrospective single center data analysis was performed.

### Surgical technique

**Figure 1:**
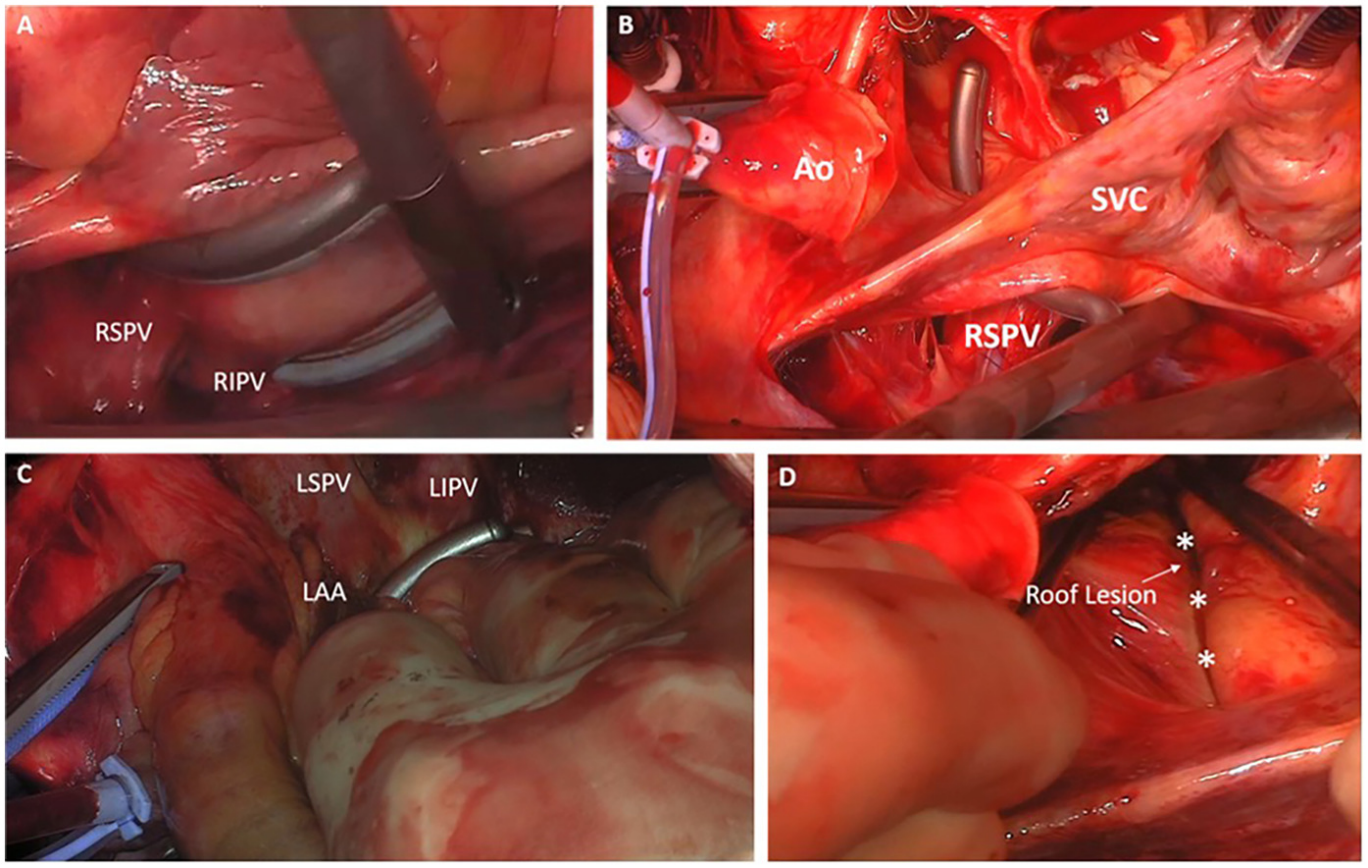
Creation of the left-atrial box lesion with the bipolar radio-frequency clamp. **(A)** Introduction of the curved bipolar radio-frequency clamp from the right-sided pulmonary veins **(B**,**C)** passing of the clamp to the side of the left pulmonary veins and ablation of the box lesion **(D)** LA roof lesion after the ablation procedure (*Roof line).

**Figure 2:**
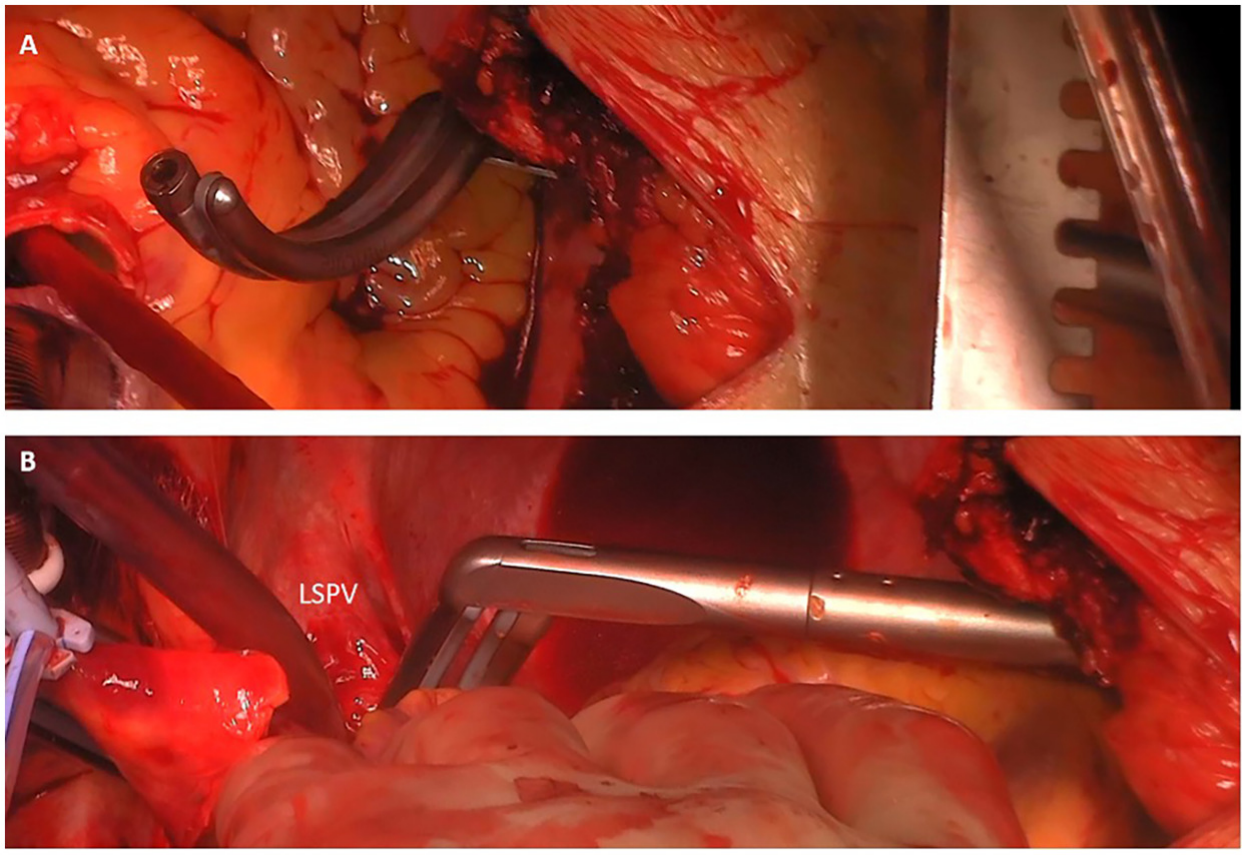
Completion of the box lesion from the left side. **(A)** Introduction of the bipolar radio-frequency clamp through the drainage tube hole. **(B)** Completion of the LA box from the left side. Introduction of the clamp from the left side. Overlapping with the previously performed ablation lines from the right side to complete the box.

**Figure 3:**
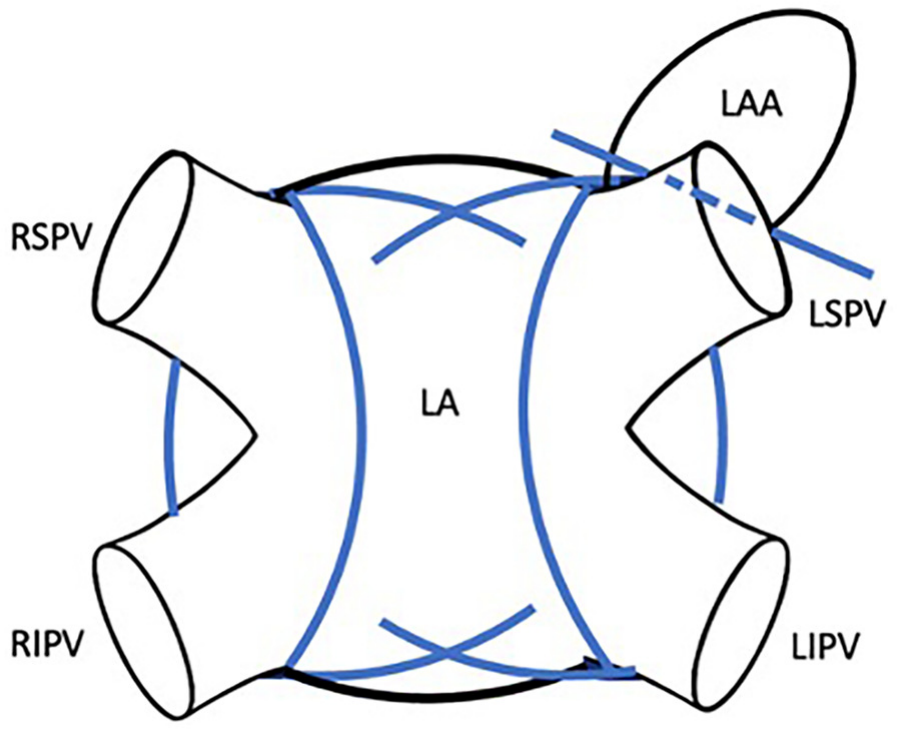
Closed-atrium box lesion set including bilateral pulmonary vein isolation, isolation of the left-atrial posterior wall and electrical isolation and occlusion of the LAA using atriclip.

All patients in this study were treated with an Atricure Isolator Synergy Access Clamp EML2, EMR2 or EMT1, (Atricure Inc. West Chester, Ohio). The curved bipolar radio-frequency clamp was introduced from the right-sided pulmonary veins through the transverse sinus to the end of the left-sided pulmonary veins (Figure 1). In case of enlarged atria, the box lesion was completed from the left side (Figure 2). To have the right angle to complete the box lesion from the left side, the radio-frequency clamp was introduced through the left-sided drainage tube hole (Figure 2). Alternatively, after opening of the oblique and transverse sinus, the curved bipolar radio-frequency clamp was introduced from the right side and subsequently from the left side to complete the left atrial box. The lesion set results in a bilateral epicardial isolation of the pulmonary veins and the left atrial posterior wall. Additionally, the Atriclip provided electrical isolation of the LAA: (Figure 3). The electrical isolation of the box was confirmed using the Atricure Isolator MAX Pen 5 (Atricure Inc. West Chester, Ohio). All patients received LAA closure, using the Atriclip or Atriclip PRO1 40 or 45 mm (Atricure Inc. West Chester, Ohio). Intraoperative TEE was used to confirm successful occlusion of the left left atrial appendage.

### Follow-up

24-h Holter ECG was conducted at latest follow-up. AF recurrence was defined by AF episode >30 s in 24 h- Holter-ECG. The discharge rhythm results were obtained by 12 lead ECG. Anticoagulation regimen was maintained for 3 months postoperative in all patients and then adapted according to CHA_2_DS_2_-VASc Score. Patients undergoing biological AVR received either coumadin, or NOAC according to their preoperative medication. In CABG patients, thrombocyte aggregation with Clopidogrel was added to the pre-operative anticoagulation (NOAC or Coumadin) for 6 months.

In patients without contraindications, amiodarone was used as first-line antiarrhythmic drug therapy, otherwise other class I or III antiarrhythmic drugs were used for at least 3 months postoperative.

### Statistical analysis

All statistical analyses were performed with SPSS statistical software version 21.0 (SPSS Inc., Chicago, Illinois) Continuous values are expressed as mean ± standard deviation Categorical variables are displayed as frequency and percentages.

## Results

### Patient characteristics

The study population consisted of 22 patients with a mean age of 67.9 ± 5.2 years. Of these, 15 patients (68.2%) were male. Median CHA₂DS₂-VASc Score was 3.0 (IQR 2;4). Paroxysmal atrial fibrillation (AF) was observed in 10 patients (45.5%), while 12 patients (54.5%) had persistent or long-standing persistent AF. The mean left atrial (LA) volume index was 41.8 ± 7.7 ml/m^2^ and the average AF duration was 3.1 ± 2.7 years. The mean left ventricular ejection fraction (LVEF) was 54.7 ± 6.2%.

Comorbidities were prevalent, with arterial hypertension present in 13 patients (59.1%), coronary artery disease in 11 (50.0%), hyperlipidemia in 6 (27.3%), and diabetes mellitus in 5 (22.7%). Chronic kidney disease was documented in 5 patients (22.7%), and 2 patients (9.1%) had a history of stroke. One patient (4.6%) had a preoperative pacemaker (Table 1).

**Table 1:** Patient baseline characteristics.

Patients	*n* = 22
Age (years)	67.9 ± 5.2
Sex [male *n*, (%)]	15 (68.2)
Type of Atrial fibrillation - Paroxysmal *n* (%) - Persistent/long-standing persistent *n* (%)	10 (45.5) 12 (54.5)
La volume index (ml/m^2^)	41.8 ± 7.7
AF duration (years)	3.1. ± 2.7
LVEF (%)	54.7 ± 6.2
Diabetes mellitus *n* (%)	5 (22.7)
Hyperlipidemia	6 (27.3)
Arterial hypertension *n* (%)	13 (59.1)
Chronic kidney disease *n* (%)	5 (22.7)
Coronary artery disease *n* (%)	11 (50.0)
Previous stroke *n* (%)	2 (9.1)
Preoperative pacemaker (%)	1 (4.6)

### Procedural data

Performed surgical procedures were isolated coronary artery bypass grafting (CABG) in 5 (22.7%) patients. Aortic valve replacement was performed in 14 (63.6%) patients. A combined CABG and AVR operation was conducted in 3 (13.6%) patients.

Mean cross-clamp time was 58.4 ± 6.3 min, mean cardiopulmonary bypass time was 93.3 ± 14.3 min.

No major ablation related complication occurred in any of the patients. There was no intraoperative death and no in-hospital mortality. One patient received re-thoracotomy for bleeding, unrelated to the performed ablation. There was no intra- or perioperative stroke in any of the patients. Permanent Pacemaker implantation was necessary in one patient (4.6%) during follow-up (Table 2).

**Table 2:** Perioperative data.

Patients	*n* = 22
CABG *n* (%)	5 (22.7)
AVR *n* (%)	14 (63.6)
CABG + AVR *n* (%)	3 (13.6)
Mean cross clamp time (min)	58.4 min ± 6.3
Mean cardiopulmonary bypass time (min)	93.3 ± 14.3
Successful LAA closure (TEE) *n* (%)	22 (100)
Re-thoracotomy for bleeding *n* (%)	1 (4.6)
In-hospital mortality *n* (%)	0 (0)
Perioperative stroke *n* (%)	0 (0)
Postoperative pacemaker implantation *n* (%)	1 (4.6)

### Rhythm results

Acute epicardial entrance- and exit block was confirmed intraoperatively in 21/22 patients, One patient that was still in SR after cross clamp release and intraoperative electrical cardioversion only had entrance block documented. Freedom from AF at discharge was obtained by 12-lead ECG and was 86.4%. All patients received 24 h Holter-ECG at latest follow-up (mean follow-up duration 12.6 ± 3.9 months). The rate of freedom from AF at latest follow-up was 77.2%, while freedom from AF off antiarrhythmic drugs was 68.2%, respectively.

Intraoperative transesophageal echo confirmed successful LAA closure in all patients, without any leakage or stump >10 mm.

## Discussion

In this study, we demonstrated the safety and efficacy of a novel technique for performing a closed atrium bipolar radio-frequency box lesion, which isolates the posterior left atrial wall. No ablation-related complications were observed, and freedom from atrial fibrillation (AF) was achieved in 77.2% of patients at a mean follow-up of 12.6 ± 3.9 months. The box ablation can be achieved without need for additional tools.

In a landmark electrophysiological study by Haissaguerre et al., it was shown that in cases of stand-alone paroxysmal AF, the triggers initiating AF predominantly originate from the pulmonary veins (1). Since then, catheter-based ablation strategies have primarily targeted the pulmonary veins (2, 3). In surgical AF ablation, the Cox-Maze procedure—first performed by James Cox in 1987—has remained the gold standard, with excellent outcomes. However, it requires opening both atria for ablation (4). Over the years, several modifications to the biatrial lesion set have been proposed (5). In patients with paroxysmal AF, a left atrial lesion set or isolated pulmonary vein isolation (PVI) are commonly employed alternatives (6–9).

In cases involving concomitant coronary artery bypass grafting (CABG) or aortic valve replacement (AVR), many surgeons are reluctant to open the left atrium for surgical ablation due to the increased cross-clamp and cardiopulmonary bypass times, as well as added procedural complexity. Whether the addition of a complete left atrial lesion set improves rhythm outcomes remains a subject of debate. In recent years, only a limited number of studies have compared complete left atrial lesion sets with PVI alone, with conflicting results. A recently published randomized controlled trial investigating surgical AF ablation during mitral valve surgery found that isolated PVI achieved similar results to a biatrial lesion set (10). In contrast, Soni et al. (11) demonstrated that a complete left atrial lesion set significantly improved freedom from AF compared to PVI alone—reporting rates of 57% in the PVI group vs. 76% in the extended left atrial group (*p* < 0.001).

Furthermore, Henn et al. showed that isolating the posterior left atrial wall resulted in significantly better long-term outcomes. At 1-year follow-up, sinus rhythm was observed in 93% of patients with box isolation and 85% without. However, at 5-year follow-up, sinus rhythm was maintained in 78% of the box isolation group compared to only 45% of the group without posterior wall isolation (12).

Our new technique for a closed atrium bipolar radio-frequency box lesion provides a safe and straightforward method to isolate both the pulmonary veins and the posterior left atrial wall. Importantly, the procedure does not require atriotomy, thereby simplifying the operation and reducing cross-clamp time, as the ablation can be performed on the beating heart. After opening the oblique and transverse sinuses, the bipolar radio-frequency clamp can be inserted from the right-sided to the left-sided pulmonary veins. In cases of significantly enlarged left atria, the box lesion can be completed from the left side by introducing the clamp through the left-sided drainage tube incision to achieve an optimal angle. This approach is technically simple and was not associated with any procedural complications in our series.

The ablation was performed on cardiopulmonary bypass without aortic cross-clamping in 82% of patients. In four cases, cross-clamping and cardiac arrest were required due to markedly enlarged left atria, which made it difficult to access the left pulmonary veins via the transverse and oblique sinuses from the right side. The use of a bipolar clamp for all lesions is a more reliable method for ensuring complete transmurality compared to surgical ablation with unipolar devices (13).

We observed a low rate of postoperative permanent pacemaker implantation (4.6%), which is comparable to previously published findings (11).

Our freedom from AF rate of 77.2% at a mean follow-up of 12.9 months is consistent with previous studies. For example, Soni et al. (11) reported a sinus rhythm rate of 76% at 1 year, which is comparable to our data.

In patients with long-standing persistent AF, a more extensive lesion set—such as a complete left atrial or even biatrial ablation—may be necessary to effectively eliminate non-pulmonary vein triggers and rotors. Additionally, in patients with structural mitral valve disease, AF may originate from the left atrial wall itself due to increased atrial volume and pressure caused by mitral regurgitation. In such cases, a more comprehensive lesion set, including a mitral isthmus line and possibly right atrial lesions, may be required.

Alternatively, hybrid approaches combining epicardial surgical and endocardial catheter-based ablation have gained increasing popularity, particularly in patients with symptomatic persistent or longstanding persistent atrial fibrillation (AF). Hybrid ablation has received a Class IIa recommendation in the most recent ESC/EACTS/EHRA guidelines (14). Recent studies have demonstrated promising outcomes in this patient population, reporting 12-month freedom from AF rates between 67% and 71.9% (15–17). Various surgical access routes—including thoracoscopic and robot-assisted techniques—have been employed in combination with endocardial catheter ablation (15–17).

In contrast to our patient cohort, these hybrid ablation studies included only patients with lone AF and without significant structural heart disease, thereby limiting direct comparability. Nonetheless, catheter-based endocardial ablation remains a valuable therapeutic option in our population, particularly in the event of symptom recurrence or AF recurrence following the blanking period. In such cases, a second-step endocardial procedure to identify conduction gaps or additional triggers for AF may be warranted and should be considered as part of a stepwise rhythm control strategy.

Future studies with longer follow-up periods and larger patient cohorts are needed to validate our preliminary findings with this novel closed atrium bipolar radio-frequency box lesion technique.

## Limitations

This study is a retrospective study with the potential risk of bias by unknown confounders. Furthermore, our series is a single-center study and is limited by the small number of patients and rather short follow-up period.

## Data Availability

The original contributions presented in the study are included in the article/Supplementary Material, further inquiries can be directed to the corresponding author.
