# Supplementary material for: Closed atrium bipolar radiofrequency box lesion for concomitant surgical atrial fibrillation ablation
Source: Front Cardiovasc Med. 2025 Aug 20;12:1655695. doi: 10.3389/fcvm.2025.1655695 (PMC12406703; doi:10.3389/fcvm.2025.1655695)
Supplement: Supplementary file 1 [file Image1.pdf]

Closed atrium bipolar radiofrequency box lesion for concomitant surgical atrial fibrillation ablation

Evaluation of a novel closed atrium bipolar radiofrequency left-atrial box ablation technique (n=22).
Mean follow-up 12.6 ± 3.9 months

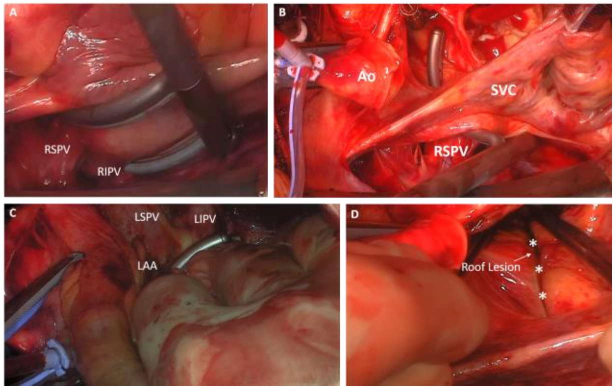

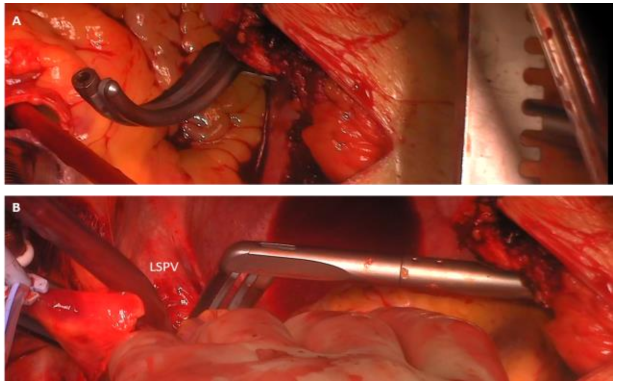

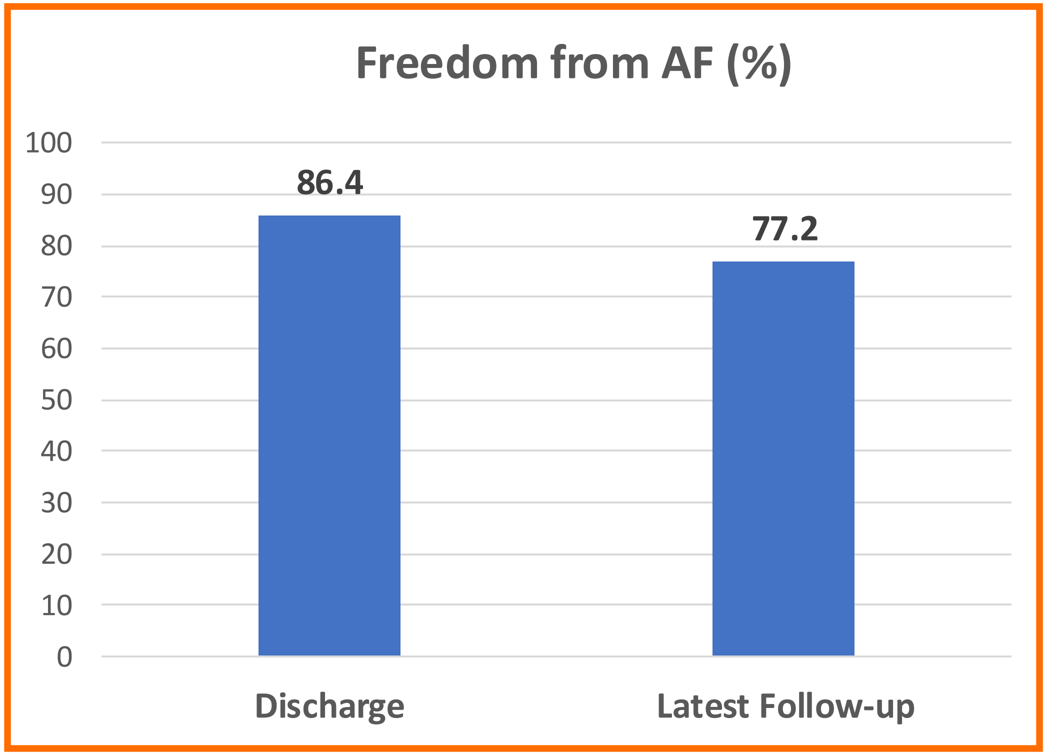

The closed atrium left atrial box lesion technique for surgical treatment of AF concomitant with CABG or AVR is safe and technically feasible. This approach enables complete isolation of the posterior left atrial wall without the need to open the left atrium.
